# Determinants and Projections of Minimum Acceptable Diet among Children Aged 6–23 Months: A National and Subnational Inequality Assessment in Bangladesh

**DOI:** 10.3390/ijerph20032010

**Published:** 2023-01-21

**Authors:** Md. Shafiur Rahman, Md. Rocky Khan Chowdhury, Md. Rashedul Islam, Sarah Krull Abe, Kamal Hossain, Toshiki Iwabuchi, Kenji J. Tsuchiya, Stuart Gilmour

**Affiliations:** 1 Research Centre for Child Mental Development, Hamamatsu University School of Medicine, Hamamatsu 431-3192, Japan; 2 United Graduate School of Child Development, Osaka University, Kanazawa University, Hamamatsu University School of Medicine, Chiba University and University of Fukui, Suita 565-0871, Japan; 3 Department of Public Health, First Capital University of Bangladesh, Chuadanga 7200, Bangladesh; 4 Department of Epidemiology and Preventive Medicine, School of Public Health and Preventive Medicine, Monash University, St. Kilda Road, Melbourne, VIC 3004, Australia; 5 Hitotsubashi Institute for Advance Study, Hitotsubashi University, Tokyo 186-8601, Japan; 6 Division of Prevention, National Cancer Center Institute for Cancer Control, Tokyo 104-0045, Japan; 7 Department of Population Science and Human Resource Development, Rajshahi University, Rajshahi 6204, Bangladesh; 8 Division of Biostatistics and Bioinformatics, Graduate School of Public Health, St. Luke’s International University, Akashi-cho, Chuo-ku, Tokyo 104-0044, Japan

**Keywords:** minimum acceptable diet, meal frequency, diet diversity, socioeconomic inequality, education-based inequality, projection, Bangladesh

## Abstract

Subnational evidence on the level of inequality in receiving complementary feeding practice among Bangladeshi children is lacking. This study estimated inequality in the minimum acceptable diet (MAD) among Bangladeshi children aged 6–23 months, and identified risk factors for and developed projections of the MAD up to 2030. Data from the Bangladesh Demographic and Health Survey 2017–2018 were used in this cross-sectional study. Regression-based slope (SII) and relative index of inequality (RII) were used to quantify the level of absolute and relative inequality, respectively. A Bayesian logistic regression model was used to identify the potential determinants of a MAD and project prevalence up to 2030. About 38% of children aged 6–23 months received a MAD. The national prevalence of a MAD was 26.0 percentage points higher among children from the richest compared to the poorest households, and 32.1 percentage points higher among children of higher-educated over illiterate mothers. Socioeconomic inequality was found to be the highest in the Chattogram division (SII: 43.9), while education-based inequality was highest in the Sylhet division (SII: 47.7). Maternal employment and the number of ANC visits were also identified as significant determinants of a MAD, and the prevalence of a MAD was projected to increase from 42.5% in 2020 to 67.9% in 2030. Approximately two out of five children received a MAD in Bangladesh and significant socioeconomic and education-based inequalities in the MAD were observed. Subnational variation in socioeconomic and education-based inequalities in the MAD requires further public health attention, and poverty reduction programs need to be strengthened.

## 1. Introduction

Weaning onto solid foods is necessary for an infant (under 12 months) from around 6 months of age by which a baby slowly gets used to eating a wider variety of foods and relies less on breast milk. Along with breastfeeding, it is essential for children until 23 months of age to gradually increase food consistency, variety of nutrition-rich foods, fortified complementary food supplements and feeding frequency according to the child’s requirement and ability [1]. Early childhood, particularly children under 2 years of age, is considered a critical window for cognitive development and physical transition [2]. Adequate feeding practice plays a significant role in supporting this development. Inadequate and inappropriate feeding practice during the first two years of life leads to micronutrient deficiency and childhood undernutrition [3]. The risks of mortality [4] and morbidity also increase [5]. Evidence suggests that almost 25% of child mortality could be prevented by achieving a universal coverage of nutritional interventions including complementary feeding practice, breastfeeding and provision of vitamin A and zinc supplementation [6]. In this context, insufficient complementary feeding practice (CFP) remains a public health issue particularly in low- and middle-income countries (LMICs). Globally, in 2020, an estimated 149.2 million children under five were stunted, 45.4 million were wasted and 38.9 million were underweight, of which a majority were from LMICs [7].

The World Health Organization (WHO) recommends ensuring a minimum acceptable diet (MAD) for all children, particularly in the early years, to promote healthy growth and development. However, based on surveys conducted in 80 LMICs between 2010–2019, the MAD was not attained among 50% of children in more than 90% of LMICs [8]. Particularly in South Asia, timely complementary feeding started only among 54.7% of children and the MAD was attained only among 20.5% of the total young children [8]. As a result, South Asian countries carry the higher burden of childhood malnutrition. The prevalence of stunting was over 30% in Afghanistan, Bangladesh, Nepal and Pakistan [7]. Despite significant improvements in nutritional interventions to support infant and young child feeding (IYCF), the prevalence of undernutrition is still high (22% in 2017) in Bangladesh [9]. The government of Bangladesh (GoB) has set a national target, as part of the 4th Health, Population and Nutrition Sector Program (4th HPNSP) of the Ministry of Health Family Welfare (MOHFW), to increase the proportion of children aged 6–23 months fed a minimum acceptable diet to 45% by 2022 [10]. The achievement of a MAD among young children is crucial for achieving global nutrition targets; the Global Nutrition Target 2025 [11] and the Sustainable Development Goal (SDG) target 2.2 state to “end all forms of child malnutrition by 2030” [12].

Overall, weaning practice among young children is still lower in Bangladesh [13]. Prior evidence suggests that the level of complementary feeding practice (CFP) in Bangladesh is higher among children from the wealthiest families [8,14]. Substantial variation exists in CFP sufficiency [14], highlighting the need for inequality assessment at subnational levels. Previous studies only examined socioeconomic inequalities in complementary feeding practice at the national level. These studies did not explore the level of inequality based on other important dimensions such as maternal education and living area (urban or rural) [8]. Although socio-economic inequalities in maternal and child health-related indicators in Bangladesh are well documented [15,16,17], evidence on such inequalities in IYCF practices is limited. Inequality assessment by region, household socioeconomic status, maternal education and rural or urban residence could provide valuable information for designing appropriate policies. Based on previous studies on maternal and child health and nutrition-related indicators, we hypothesize that the magnitude of inequality in IYCF indicators will vary across regions [8,14,17,18]. A comprehensive assessment of inequality in feeding practices, based on different dimensions at the subnational level in Bangladesh, is essential for supporting the development of policies and reducing inequality. Moreover, it is critical to identify risk factors associated with feeding practices to minimize the future burden of malnutrition.

Therefore, this study aimed to quantify the level of socioeconomic and education-based absolute and relative inequalities in minimum dietary diversity (MDD), minimum meal frequency (MMF) and the MAD among Bangladeshi children aged 6–23 months at the subnational level. This study also identified the potential determinants of a MAD and calibrated projections of the MAD up to 2030.

## 2. Materials and Methods

### 2.1. Data

This study used cross-sectional data from the Bangladesh Demographic and Health Survey (BDHS), a nationally representative population-based household survey, conducted from 2017 to 2018 [9]. Households in the BDHS were selected using a multistage stratified sampling technique. In the first stage, 675 primary sampling units (PSUs), based on enumeration areas (clusters) from the census survey 2011 designed by the Bangladesh Bureau of Statistics, were selected with probability proportional to size [9]. Finally, a total of 30 households per PSU were selected with equal probability. Details of the survey methodology and questionnaire are discussed elsewhere [9].

### 2.2. Study Participants

Children aged 6–23 months with available information on IYCF were included in this study. Out of 2474 eligible children aged 6–23 months, 47 were excluded as they were not the youngest child (Figure S1). Finally, a total of 2427 children aged 6–23 months were included in this study.

### 2.3. Outcome Measures

The main outcomes of interest were the percentage of MDD, MMF and the MAD. The MMF was defined as the proportion of children aged 6–23 months that received solid, semi-solid or soft foods at least the minimum number of recommended times in the past 24 h (i.e., 2 times for breastfed infants aged 6–8 months, 3 times for breastfed children aged 9–23 months and 4 times for non-breastfed children aged 6–23 months), as reported by their mothers [19]. Similarly, information on children’s consumption of food from seven groups (Table S1) was collected to estimate the proportion of MDD. A child was considered as having MDD if the child received food items from at least four food groups in the past 24 h [19]. Then, the proportion of a MAD was estimated as the proportion of children who received both the minimum number of meals and had MDD in the past 24 h [19].

### 2.4. Covariates

Following previous studies [20,21,22,23], variables representing the child and Mothers’ characteristics and household and community characteristics were included in this study. Child characteristics included age group (6–11, 12–17 and 18–23 months), sex, birth order (first, second, third or higher), child underweight (defined as weight-for-age being -2SD below the reference population; no, yes) and stunting (defined as height-for-age being -2SD below the reference population; no, yes). Mothers’ characteristics were mother’s age (years), mother’s educational status (no education, primary, secondary and higher), mother’s working status (currently working, not working), access to mass media (not at all, yes—at least to some extent) and the number of antenatal care (ANC) visits (no ANC visit, 1–3 visits, 4 or above). Household characteristics included wealth quintile (poorest, poorer, middle, richer, richest) and sex of household head (male, female). Community characteristics were place of residence (urban, rural) and division (Barisal, Chattogram, Dhaka, Khulna, Mymensingh, Rajshahi, Rangpur, Sylhet). Wealth quintile was used as a proxy variable to measure socioeconomic status in the absence of information on income of households. Wealth index in the DHS survey is calculated by the survey administrator, based on information on household characteristics and assets using principal component analysis [24]. Then, households are classified into quintiles based on the values of the wealth index, where households with lower values of the index are considered as the poorest and vice versa.

### 2.5. Statistical Analysis

The percentages of MDD, MMF and the MAD along with 95% confidence intervals (CI) were estimated after adjusting for sampling weight. Regression-based slope index of inequality (SII) and relative index of inequality (RII) were used to measure the magnitude of absolute and relative inequality, respectively, in the percentages of MMF, MDD and the MAD. Socioeconomic and education-based inequalities were assessed using the SII and RII, at the national level, across urban and rural areas and across regions (divisions). The SII presents the absolute difference in the percentage points in the proportion of children receiving MMF, MDD and the MAD between the top and bottom quintile/class of socioeconomic or educational status, whereas the RII presents the ratio of the children receiving MMF, MDD and the MAD between the top and bottom class of socioeconomic or educational status.

A multivariable logistic regression model was used to identify potential determinants of a MAD. Both unadjusted and adjusted odds ratios (ORs) and 95% CI were reported. All of the logistic regression models and inequality analyses accounted for sampling weights. In addition, a Bayesian linear regression analysis with year as a covariate was used to develop projections of the proportion of a MAD up to 2030. Summarized data on the MAD in 2011, 2014 and 2017–2018 were used to develop projections up to the year 2030 on the assumption that there would be no major changes in the nutrition policy [9,25]. Models were performed on the logit transformed values and then results were transformed back in order to ensure that the projected values lay between 0 and 1. Non-informative priors were assigned to regression coefficients and precision. A Markov chain Monte Carlo (MCMC) algorithm was used to generate samples from the posterior distribution using two chains, where the first 5000 samples were discarded as burn-ins and then 1000 samples from each chain were obtained using 30,000 iterations with a thinning rate of 30. Convergence was diagnosed visually using trace plots (Figure S2) and quantitatively analyzed using Gelman–Rubin diagnostic statistics. Finally, mean value and 95% credible intervals were estimated. Data management was performed in Stata MP 16.1 and statistical analyses were performed in Stata (inequality assessment and determinant analysis), R and JAGS (Bayesian models).

## 3. Results

### 3.1. Participant Characteristics

A total of 2427 children aged 6–23 months were included, of whom 51.7% were boys and 37.4% were first-born children (Table 1). About 18% of children were underweight. The majority of the children (75.2%) were born to women aged 20–35 years, while 18.4% were born to adolescent mothers aged <20 years. About one quarter (27.4%) of mothers were primary educated. One third (33.3%) of the children were from urban areas (Table 1).

### 3.2. Prevalence of a MAD

**Figure 1:**
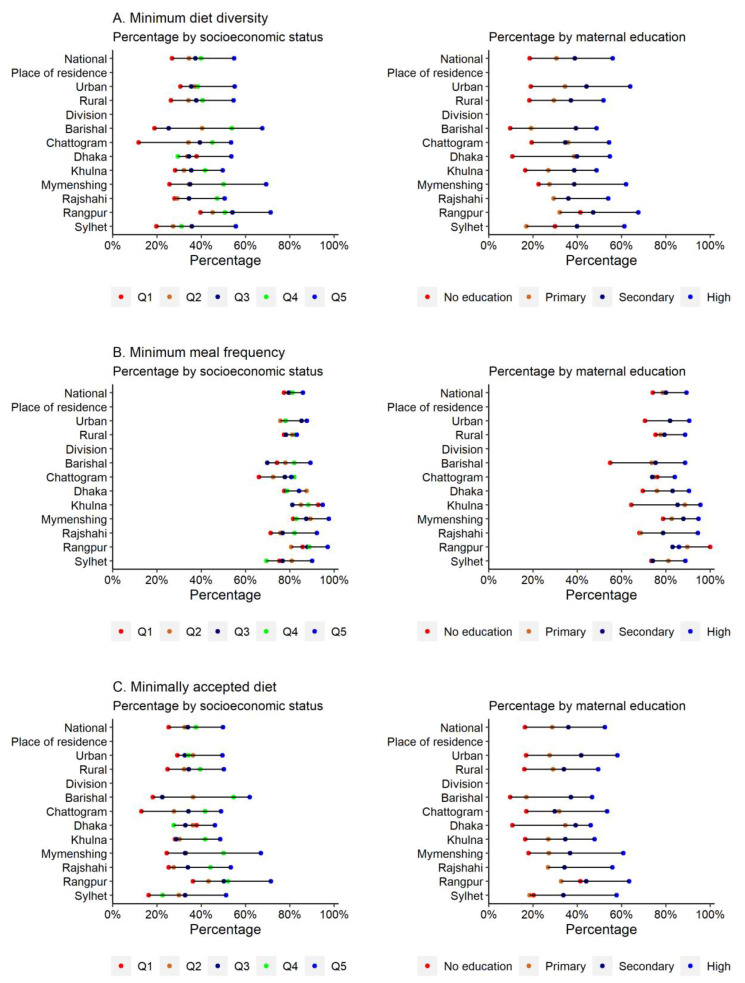
Prevalence of minimum diet diversity, minimum diet frequency and minimally accepted diet among Bangladeshi children aged 6–23 months by household socioeconomic status and maternal educational status.

The proportion of Bangladeshi children aged 6–23 months attaining the MDD, MMF and MAD thresholds were 38%, 81% and 36%, respectively (Table 1). A substantially lower prevalence of MDD was observed among children from the poorest households (26.7%), children of illiterate mothers (18.5%) and those with mothers who lacked access to mass media (30.5%). In comparison, the prevalence of MMF was higher among children of mothers who had access to mass media (82.9%) and received ≥4 ANC visits (85.3%). The proportion of children receiving a MAD was lower for children aged 6–11 months (21.7%), children from the poorest households (29.3%) and children of illiterate mothers (16%) and mothers who did not have ANC visits (20.7%). At the subnational level, the Rangpur division had the highest prevalence of a MAD (44%), whereas Sylhet had the lowest prevalence (28%). Children from the richest households in the Rangpur division had the highest prevalence of a MAD followed by Mymenshing and Barishal, while the lowest prevalence was observed among children from the poorest households in Chittagong followed by Sylhet (Figure 1, Table S2). Similarly, only 20% of children of non-educated mothers from Barishal, Dhaka and the Sylhet division received a MAD (Figure 1, Table S3).

### 3.3. Absolute Inequality in the MAD

The slope index of inequality exhibited relatively higher levels of socioeconomic inequality in the prevalence of MDD (SII: 29.8) and MAD (26.0) at the national level, indicating that the prevalence of MDD and MAD are 29.8 percentage points and 26.0 percentage points higher, respectively, among children from the richest households compared to children from the poorest households (Table 2). At the subnational level, the highest level of absolute socioeconomic inequality was observed in the Chattogram division (43.9), followed by Sylhet (39.9) and Barishal (35.7). Similarly, the prevalence of MDD and MAD were 34.3 percentage points and 32.1 percentage points higher, respectively, among children of higher-educated mothers than children of illiterate mothers (Table 2). Education-based inequality in the prevalence of a MAD was found to be the highest in Sylhet (47.7) followed by Barishal (40.7). In addition, large socioeconomic and maternal education-based absolute inequality was observed for the consumption of dairy products among children at the national level (Tables S4 and S5).

### 3.4. Relative Inequality in the MAD

Nationally, children from the richest households were 2.10 times more likely to receive a MAD compared to their poor counterparts (Table S6). Similarly, children of highly educated mothers were 2.53 times more likely to receive a MAD than children of illiterate mothers. Children from rich households in Sylhet (RII: 3.84), Chattogram (3.79) and the Barishal division (3.15) were more than three times more likely to receive a MAD than their poor counterparts. Similarly, the magnitude of education-based relative inequality was found to be the highest in the Sylhet division (5.23), whereas the magnitude was the lowest in the Dhaka division (2.12) (Table S6).

### 3.5. Determinants of a MAD

Multivariable logistic regression identified child’s age, mother’s education, working status, ANC visit and higher socioeconomic status as significant determinants of a MAD (Table 3). Children whose mother received four or more ANC visits during the pregnancy had 2.30-times-higher odds (OR: 2.30; 95% CI: 1.51–3.51) of receiving a MAD compared to children whose mother did not have ANC visits during pregnancy.

### 3.6. Trends and Future Directions of the MAD

**Figure 2:**
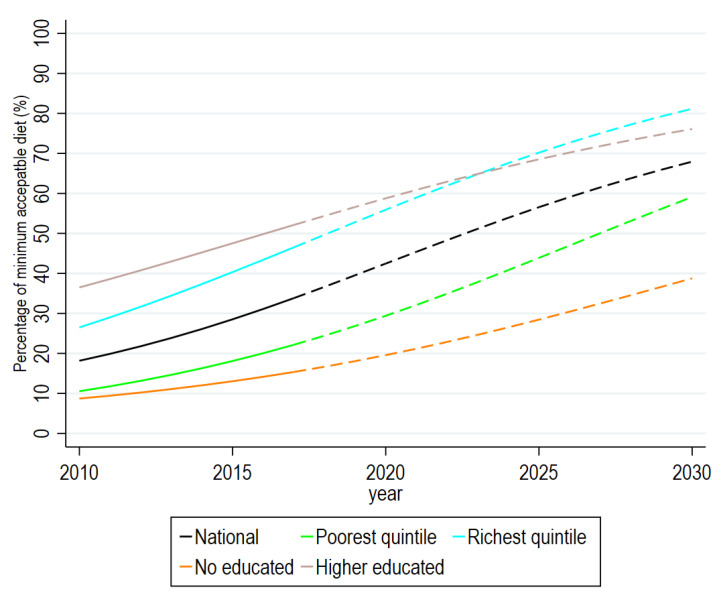
Adjusted prevalence of minimum diet diversity, minimum diet frequency and minimally accepted diet among Bangladeshi children aged 6–23 months. Note: Models were adjusted by age and sex of child, and age of mother. Exact prevalence along with 95% confidence intervals are presented in Table S7.

If the current trends continue, the prevalence of a MAD is projected to increase from 42.5% (CrI: 21.6–63.4) in 2020 to 48.3% (CrI: 22.5–74.1) by 2022 and 67.9% (CrI: 21.1–95.6) by 2030, respectively (Figure 2, Table S8). Similarly, 62% of children from the richest households are expected to receive a MAD by 2022, while such a proportion is projected to increase to 81.2% by 2030.

## 4. Discussion

To the best of our knowledge, this is the first subnational-level study to evaluate the magnitude of socioeconomic and education-based inequality in the prevalence of MDD, MMF and MAD among younger children in Bangladesh. Substantially higher levels of socioeconomic and education-based inequality in the prevalence of MDD and MAD were observed at the national level with even higher levels of inequality in urban areas. At the subnational level, considerably higher levels of pro-rich and education-based inequality were observed in the Sylhet, Barishal and Chattogram divisions. In addition, maternal employment situation and number of ANC visits during pregnancy, along with household socioeconomic status and maternal education, were identified as significant factors affecting the uptake of a MAD. If the current trends continue, about 32% of younger children in Bangladesh will fail to receive a MAD by 2030.

The rate of receiving the MMF among younger children was more than 80% in Bangladesh while less than half of the children were receiving MDD and a MAD. The present study observed that the proportion of children receiving a MAD in Bangladesh increased in the past decade (from 21% in 2011 to 38% in 2018) [9,25]. In neighboring countries, such rates varied from 15% in India to 71% in Sri Lanka [26]. Substantial variation in the proportion of children receiving a MAD was observed across subnational levels; the proportion receiving a MAD was found to be highest in the northwestern region (Rangpur division) and lowest in the northeastern region (Sylhet division). The exact reason for these regional variations in the MAD is unknown, but is likely due to poverty, lower levels of education and lack of awareness on feeding patterns in poorer rural areas [27,28]. Women and children in these regions, especially in remote rural areas of these regions, remain isolated with poor communication and transportation. This leads to limited access to higher education and employment, and an inability to allocate budget to nutritious foods [17,29,30]. On the other hand, the northwestern division was more pronounced in achieving a higher preschool attendance rate and had better performance in meeting early childhood development index targets compared to the northeastern and southern region [31]. Over the past few decades, many developmental initiatives have been introduced by the government, as well as by local and international organizations, to reduce poverty and improve maternal and child health, which may have helped this region to improve complementary feeding practice as well as to reduce child malnutrition. NGO-driven initiatives such as community-based counseling on dietary diversity and cooking demonstrations in this region also helped to improve mothers’ knowledge on their children’s diets [32,33,34,35]. Regional differences in receiving a MAD may halt the national-level progress in increasing the overall rate of the MAD. Therefore, policy makers must consider regional variation in dietary patterns while designing national nutrition programs.

Although around half of the children from the richest households received a MAD at the national level, only a quarter of children from the poorest households received a MAD. Such pro-rich inequality was even higher among urban residents compared to their rural counterparts. This finding is consistent with a prior study conducted in India [32]. The overall proportion of children receiving a MAD in rural areas was low. Moreover, variation in such proportions across children from households with different socioeconomic conditions was also lower. These findings highlight the need for prioritization of interventions among rural children irrespective of their SES, as well as among children from poor households in urban areas. Furthermore, at the subnational level, relatively higher levels of inequalities were observed in the southeastern region (Chittagong) and north-eastern region (Sylhet). The subnational variations in socioeconomic inequality observed in our study somewhat corroborate prior studies conducted in Bangladesh [17,30,36]. Despite having lower poverty rates in the eastern part of Bangladesh, poor child nutritional status in this region might be due to unique environmental characteristics of some parts of this region. The Chittagong region has many hill tracks and coastal belts, and the Sylhet region has the *Haor* basin—a unique wetland geography (ox-box lake) requiring specialized agricultural practices [37]. People living in the hill tracts of Bangladesh are very vulnerable, due to low income and few employment opportunities, limited access to health care and other social services [38]. On the other hand, the people living in different *Haor* areas often suffer from food insecurity due to pre-monsoon flash floods and single crop production [37,39]. These adversities may affect complementary feeding practice, particularly because often women from such areas participate in agricultural activities or income-generating activities [40]. The expansion of employment opportunities and ensuring food security for the very poor might reduce the socioeconomic gap in dietary patterns among younger children. These inequalities in basic social needs such as the MAD also show the importance of poverty alleviation in Bangladesh and the formation of sustainable economic systems that enable all people in the country to afford to live a life of basic dignity.

This study found that children of higher-educated mothers were more likely to receive a MAD than children of mothers with no formal education, which is consistent with previous studies conducted in Afghanistan and Nepal [23,41]. Similar to socioeconomic inequality, education-based inequality in receiving a MAD was higher in the northeastern region (Sylhet division). Increasing the female literacy rate, while reducing the costs associated with education, and poverty alleviation strategies for the poorest community members might help to increase the uptake of a MAD. A wider coverage of non-formal education schemes under NGOs and community initiatives in line with the state’s role of providing education, which have been recognized as successful mechanisms in many developing countries, might help to educate excluded people in Bangladesh [42,43].

The present study projected that approximately 68% of children will receive a MAD by 2030 in the current trend scenario, with even lower rates among children of uneducated mothers (23%) and those from the poorest families (35%), indicating that more than one third of Bangladeshi children might not receive the recommended level of an acceptable diet by 2030, despite the country projecting to achieve the national level target for the MAD (48% by 2022), ensuring that a MAD for all children from each region or community remains a challenge. Such challenges will also become a major obstacle for reaching other interrelated SDG targets, such as a reduction in undernutrition or infectious diseases [44,45]. As children who do not receive a MAD are often from the same community, identification of such communities or clusters is essential and community-based tailored nutritional interventions must be scaled up in such communities to increase the uptake of the MAD. In addition, interventions aimed at improving parents’ level of knowledge about complementary feeding and dietary requirements for children should be introduced [46]. Providing quality and appropriate counseling to mothers and caregivers by trained health workers at health facilities and at the community level might also improve the feeding practices of younger children as it was found to be very successful in Ethiopia and Vietnam [47]. It is evident that community-based approaches to the utilization of locally produced foods can meet the dietary needs of children in poor or food-insecure settings [48]. Therefore, further initiatives must be developed by the government of Bangladesh, such as setting short-term targets to enhance the IYCF practice through educational and nutritional intervention programs. Finally, the government of Bangladesh needs to revisit poverty alleviation programs and develop new, sustainable and effective strategies to lift the poorest citizens out of absolute poverty.

This study has several strengths, including its large sample size, and application of appropriate methodologies for evaluating the level and magnitude of inequality at the subnational level. The study also has a few limitations that must be mentioned. First, due to the lack of necessary information, we could not adjust for some important covariates such as household food security and parental behaviors. Future studies should adjust for these factors to precisely estimate the impact of SES and educational status on the uptake of the MAD. Second, the cross-sectional nature of the data did not establish a causal relationship between risk factors and the MAD. Third, we cannot rule out the possibility of recall bias or socially desirable bias, which may have resulted from collecting information about self-reporting age, education, occupation and household assets, although the DHS survey uses a structured and validated questionnaire.

## 5. Conclusions

Approximately two out of five young children in Bangladesh have a MAD. The magnitude of socioeconomic inequality in receiving a MAD was higher than the relative and absolute gap due to maternal education. Socioeconomic and education-based inequalities in the MAD were found to be highest in the eastern region of Bangladesh. Subnational variation in socioeconomic and education-based inequalities in the MAD requires further public health attention, and poverty reduction programs need to be strengthened. The failure to do so will ultimately harm the timely achievement of the SDG target 2.2—‘end hunger, achieve food security and improved nutrition and promote sustainable agriculture’.

## Figures and Tables

**Table 1:** Weighted percentage of minimum diet diversity, minimum diet frequency and minimally accepted diet among Bangladeshi children aged 6–23 months (N = 2427).

Characteristics	N (%)	Percentage (95% Confidence Intervals)		
		Minimum Diet Diversity	Minimum Meal Frequency	Minimally Accepted Diet
*Child characteristics*				
Age group (months)				
6–11	800 (33.0)	22.7 (20.0–25.7)	73.0 (69.8–75.9)	21.7 (19.0–24.7)
12–17	847 (34.9)	45.2 (41.9–48.6)	84.0 (81.4–86.3)	42.7 (39.4–46.1)
18–23	780 (32.1)	47.0 (43.6–50.5)	85.8 (83.1–88.0)	42.4 (39.0–45.9)
Sex				
Boys	1254 (51.7)	38.2 (35.6–40.9)	81.0 (78.7–83.1)	35.3 (32.7–38.0)
Girls	1173 (48.3)	38.6 (35.8–41.4)	80.9 (78.5–83.0)	36.1 (33.4–38.9)
Order of birth				
First	907 (37.4)	43.0 (39.9–46.3)	83.3 (80.7–85.6)	39.8 (36.6–43.0)
Second	817 (33.7)	37.2 (33.9–40.5)	79.4 (76.6–82.1)	34.7 (31.5–38.0)
Third or higher	703 (29.0)	33.8 (30.4–37.4)	79.6 (76.5–82.4)	31.6 (28.3–35.1)
Underweight				
No	1957 (82.1)	38.7 (36.6–40.8)	80.5 (78.8–82.2)	35.7 (33.7–37.9)
Yes	428 (18.0)	35.2 (30.7–40.0)	82.4 (78.3–85.8)	34.2 (29.8–39.0)
Stunting				
No	1626 (69.5)	38.0 (35.7–40.4)	80.6 (78.7–82.5)	35.3 (33.1–37.6)
Yes	715 (30.5)	38.4 (34.9–42.1)	81.0 (77.9–83.8)	35.9 (32.4–39.6)
Wasting				
No	2167 (92.2)	38.2 (36.2–40.3)	80.7 (79.0–82.3)	35.5 (33.5–37.5)
Yes	183 (7.8)	37.0 (30.3–44.1)	80.1 (73.7–85.3)	35.6 (29.0–42.7)
*Mothers’ characteristics*				
Mother’s age (years)				
15–20	449 (18.5)	36.2 (32.0–40.7)	79.4 (75.6–82.9)	33.5 (29.4–37.9)
20–35	1824 (75.2)	38.9 (36.7–41.1)	80.9 (79.1–82.7)	36.0 (33.9–38.3)
35–49	154 (6.4)	38.8 (31.4–46.8)	85.5 (79.0–90.2)	38.0 (30.7–46.0)
Mother’s educational status				
Not educated	144 (5.9)	18.5 (13.1–25.4)	74.1 (66.6–80.4)	16.3 (11.2–23.0)
Primary educated	664 (27.4)	30.6 (27.2–34.2)	78.6 (75.3–81.5)	28.7 (25.4–32.3)
Secondary educated	1168 (48.1)	38.8 (36.1–41.6)	80.0 (77.7–82.2)	36.0 (33.3–38.7)
Higher educated	451 (18.6)	56.0 (51.3–60.6)	89.4 (86.1–91.9)	52.4 (47.7–57.1)
Mother’s working status				
Not working	1529 (63.0)	37.7 (35.3–40.2)	78.8 (76.6–80.7)	34.4 (32.0–36.8)
Currently working	898 (37.0)	39.5 (36.4–42.7)	84.6 (82.1–86.8)	37.9 (34.8–41.1)
Access to mass media				
Not at all	893 (36.8)	30.5 (27.5–33.6)	77.3 (74.3–80.0)	27.7 (24.8–30.8)
Yes (at least to some extent)	1534 (63.2)	42.6 (40.2–45.1)	82.9 (81.0–84.7)	40.0 (37.6–42.5)
Number of ANC visits				
No ANC visits	192 (7.9)	24.1 (18.6–30.7)	68.0 (61.0–74.2)	20.7 (15.6–27.1)
1–3 visits	1081 (44.5)	30.6 (27.9–33.3)	78.7 (76.2–81.0)	28.6 (26.1–31.4)
4+ visits	1154 (47.6)	48.5 (45.6–51.4)	85.3 (83.2–87.3)	45.1 (42.3–48.1)
*Household level*				
Wealth quintile				
Q1 (poorest)	517 (21.3)	26.7 (23.0–30.7)	77.4 (73.6–80.9)	25.3 (21.7–29.3)
Q2	513 (21.1)	34.4 (30.5–38.6)	80.8 (77.2–84.0)	32.6 (28.8–36.8)
Q3	424 (17.5)	37.3 (32.9–41.9)	79.5 (75.5–83.0)	34.0 (29.8–38.5)
Q4	498 (20.5)	39.8 (35.6–44.2)	81.1 (77.4–84.3)	37.6 (33.5–42.0)
Q5 (richest)	475 (19.6)	54.9 (50.4–59.3)	86.0 (82.5–88.8)	49.9 (45.3–54.4)
*Contextual factors*				
Place of residence				
Urban	808 (33.3)	45.0 (41.2–48.9)	83.2 (80.1–85.9)	40.8 (37.0–44.6)
Rural	1619 (66.7)	36.0 (33.9–38.3)	80.1 (78.2–81.9)	33.9 (31.8–36.1)
Regions				
Barishal	262 (10.8)	34.1 (26.8–42.3)	76.5 (68.8–82.8)	32.0 (24.9–40.2)
Chattogram	394 (16.2)	37.9 (33.8–42.2)	76.3 (72.4–79.8)	34.2 (30.2–38.4)
Dhaka	355 (14.6)	40.3 (36.5–44.2)	81.6 (78.4–84.5)	37.2 (33.5–41.0)
Khulna	240 (9.9)	36.8 (30.7–43.4)	87.3 (82.1–91.1)	34.5 (28.5–41.0)
Mymensingh	294 (12.1)	36.7 (30.4–43.4)	86.3 (81.0–90.3)	35.3 (29.1–42.0)
Rajshahi	255 (10.5)	35.5 (30.1–41.2)	77.9 (72.7–82.4)	34.1 (28.8–39.8)
Rangpur	285 (11.7)	47.2 (41.4–53.1)	86.0 (81.4–89.6)	44.9 (39.2–50.8)
Sylhet	342 (14.1)	31.6 (25.4–38.5)	78.1 (71.7–83.4)	28.4 (22.5–35.1)
Overall	2427 (100.0)	38.4 (36.5–40.3)	80.9 (79.3–82.4)	35.7 (33.8–37.6)

Note: ANC, antenatal care.

**Table 2:** Socioeconomic and education-based absolute inequalities in minimum diet diversity, minimum diet frequency and minimally accepted diet among Bangladeshi children aged 6–23 months (N = 2427).

Slope Index of Inequality	Socioeconomic Inequality			Education-Based Inequality		
	Minimum Diet Diversity	Minimum Meal Frequency	Minimally Accepted Diet	Minimum Diet Diversity	Minimum Meal Frequency	Minimally Accepted Diet
National	29.8 (22.3–37.3) ***	8.4 (2.3–14.5) **	26.0 (18.7–33.3) ***	34.3 (26.4–42.1) ***	13.3 (6.6–20.0) ***	32.1 (24.4–39.9) ***
Place of residence						
Urban	37.5 (23.5–51.5) ***	17.4 (6.7–28.1) **	32.1 (18.6–45.6) ***	42.3 (29.4–55.2) ***	15.2 (2.7–27.7) *	42.9 (30.2–55.5) ***
Rural	25.6 (16.9–34.4) ***	5.7 (−2.1–13.5)	23.8 (14.9–32.7) ***	29.5 (20.1–39.0) ***	12.3 (4.2–20.4) **	27.1 (17.6–36.6) ***
Regions						
Barishal	37.7 (15.8–59.7) **	8.2 (−11.0–27.4)	35.7 (14.1–57.4) **	40.3 (22.3–58.3) ***	17.3 (−2.4–37.1)	40.7 (22.5–59.0) ***
Chattogram	47.1 (31.9–62.2) ***	18.9 (5.6–32.1) **	43.9 (28.0–59.8) ***	24.7 (4.9–44.5) *	6.7 (−7.7–21.2)	28.4 (10.1–46.7) **
Dhaka	25.1 (6.7–43.5) **	5.1 (−7.5–17.7)	14.1 (−3.2–31.5)	33.5 (15.1–51.9) ***	21.3 (5.3–37.2) **	27.6 (9.0–46.1) **
Khulna	29.5 (10.6–48.5) **	4.0 (−9.4–17.4)	29.4 (10.5–48.2) **	38.0 (16.9–59.0) ***	11.4 (−2.7–25.6)	35.1 (13.6–56.7) **
Mymensingh	33.0 (12.2–53.8) **	12.5 (0.9–24.0) *	32.2 (12.2–52.2)**	37.8 (18.9–56.8) ***	15.0 (2.8–27.2) **	36.8 (17.8–55.8) ***
Rajshahi	31.1 (11.2–51.1) **	23.2 (7.0–39.4) **	35.2 (16.7–53.7) ***	32.2 (11.8–52.6) **	27.9 (9.2–46.6)	39.2 (21.1–57.4) ***
Rangpur	24.3 (4.0–44.6) *	10.4 (−2.9–23.7)	28.8 (9.2–48.4) **	41.2 (21.8–60.6) ***	−5.3 (−18.1–7.5)	35.6 (16.3–54.9) ***
Sylhet	44.4 (28.1–60.8) ***	14.4 (−1.1–30.0)	39.9 (23.1–56.7) ***	51.5 (37.0–66.0) ***	6.6 (−7.5–20.8)	47.7 (31.9–63.5) ***

Note: *** *p* < 0.001; ** *p* < 0.01; * *p* < 0.05.

**Table 3:** Determinants of minimally accepted diet among Bangladeshi children aged 6–23 months (N = 2427).

Characteristics	Odds Ratio (95% Confidence Intervals)			
	Model 1 ^a^	Model 2 ^b^	Model 3 ^c^	Model 4 ^d^
*Child characteristics*				
Age group (months)				
6–11 (ref.)	1.00	1.00	1.00	1.00
12–17	2.42 (1.96–3.00)	2.52 (2.01–3.15)	2.52 (2.01–3.15)	2.53 (2.02–3.17)
18–23	2.56 (2.06–3.17)	2.68 (2.13–3.36)	2.70 (2.15–3.39)	2.70 (2.14–3.39)
Sex				
Boys (ref.)	1.00	1.00	1.00	1.00
Girls	1.08 (0.91–1.27)	1.07 (0.90–1.28)	1.08 (0.90–1.28)	1.08 (0.90–1.29)
Order of birth				
First	1.28 (1.06–1.56)	1.11 (0.88–1.41)	1.13 (0.89–1.43)	1.14 (0.90–1.44)
Second (ref.)	1.00	1.00	1.00	1.00
Third or higher	0.85 (0.69–1.06)	1.05 (0.82–1.34)	1.06 (0.83–1.35)	1.06 (0.83–1.35)
Underweight				
No (ref.)	1.00	1.00	1.00	1.00
Yes	0.84 (0.67–1.04)	1.07 (0.84–1.35)	1.09 (0.86–1.39)	1.09 (0.85–1.39)
*Mothers’ characteristics*				
Mother’s age (years)				
15–20 (<20) (ref.)	1.00	1.00	1.00	1.00
20–35	1.07 (0.86–1.33)	1.01 (0.77–1.32)	0.97 (0.74–1.27)	0.98 (0.75–1.29)
35–49 (≥35)	1.12 (0.77–1.64)	1.24 (0.77–2.01)	1.18 (0.73–1.91)	1.18 (0.73–1.92)
Mother’s educational status				
Not educated (ref.)	1.00	1.00	1.00	1.00
Primary educated	1.68 (1.06–2.65)	1.56 (0.97–2.51)	1.55 (0.97–2.50)	1.55 (0.96–2.50)
Secondary educated	2.60 (1.67–4.04)	2.25 (1.41–3.60)	2.15 (1.34–3.44)	2.10 (1.30–3.38)
Higher educated	5.70 (3.58–9.05)	4.24 (2.55–7.03)	3.66 (2.19–6.12)	3.47 (2.06–5.85)
Mother’s working status				
Not working (ref.)	1.00	1.00	1.00	1.00
Currently working	1.10 (0.92–1.3)	1.28 (1.06–1.54)	1.36 (1.13–1.65)	1.31 (1.08–1.59)
Access to mass media				
Not at all (ref.)	1.00	1.00	1.00	1.00
Yes (at least to some extent)	1.86 (1.55–2.22)	1.33 (1.09–1.62)	1.22 (0.98–1.51)	1.20 (0.96–1.49)
Number of ANC visits				
No ANC visits (ref.)	1.00	1.00	1.00	1.00
1–3 visits	1.88 (1.27–2.78)	1.53 (1.02–2.30)	1.49 (0.99–2.24)	1.44 (0.95–2.17)
4+ visits	4.03 (2.73–5.94)	2.66 (1.76–4.03)	2.46 (1.62–3.75)	2.30 (1.51–3.51)
*Household level*				
Wealth quintile				
Q1 (poorest) (ref.)	1.00		1.00	1.00
Q2	1.51 (1.15–1.98)		1.28 (0.96–1.71)	1.34 (1.00–1.80)
Q3	1.50 (1.13–1.99)		1.05 (0.77–1.45)	1.12 (0.81–1.55)
Q4	1.89 (1.44–2.48)		1.21 (0.88–1.66)	1.32 (0.95–1.84)
Q5 (richest)	3.43 (2.62–4.49)		1.76 (1.25–2.48)	1.95 (1.34–2.84)
*Contextual factors*				
Place of residence				
Rural (ref.)	1.00			1.00
Urban	1.48 (1.25–1.76)			1.07 (0.87–1.33)
Regions				
Barishal	0.83 (0.57–1.20)			1.11 (0.74–1.65)
Chattogram	0.94 (0.67–1.32)			1.10 (0.76–1.58)
Dhaka	1.03 (0.74–1.45)			1.02 (0.71–1.48)
Khulna (ref.)	1.00			1.00
Mymensingh	0.93 (0.65–1.33)			1.25 (0.85–1.83)
Rajshahi	0.99 (0.69–1.43)			1.11 (0.75–1.63)
Rangpur	1.51 (1.06–2.15)			1.68 (1.15–2.46)
Sylhet	0.79 (0.56–1.11)			1.04 (0.71–1.53)

Note: Based on the results of likelihood ratio tests, estimates of fixed effect logistic regression models were preferred over mixed-effect models. The results of mixed-effect logistic regression models are presented in Table S7. ^a^ Model 1, unadjusted model. ^b^ Model 2 included only children’s characteristics and their mothers’ characteristics. ^c^ Model 3 further included household-level characteristics. ^d^ Model 4 additionally included community-level variables (contextual factors).

## Data Availability

All data are publicly available through the Measure DHS website.

## Supplementary Materials

The following supporting information can be downloaded at https://www.mdpi.com/article/10.3390/ijerph20032010/s1. Figure S1: Participant selection; Figure S2: Trace plot for the predicted prevalence of minimum acceptable diet among Bangladeshi children in 2030; Table S1: Complementary good groups; Table S2: Prevalence of minimum diet diversity, minimum diet frequency, and minimum acceptable diet among Bangladeshi children aged 6–23 months by household socioeconomic status; Table S3: Prevalence of minimum diet diversity, minimum diet frequency, and minimum acceptable diet among Bangladeshi children aged 6–23 months by maternal education; Table S4: Proportion of children consumed different food groups by household socioeconomic status and inequality assessment at the national level; Table S5: Proportion of children consumed different food groups by maternal education and inequality assessment at the national level; Table S6: Socioeconomic and education-based relative inequality in minimum diet diversity, minimum diet frequency and minimum acceptable diet among Bangladeshi children aged 6–23 months; Table S7: Determinants of minimum acceptable diet among Bangladeshi children 6–23 months (mixed-effects models); Table S8: Projected prevalence of minimum acceptable diet among Bangladeshi children aged 6–23 months (2020–2030).
