# Supplementary material for: Determinants and Projections of Minimum Acceptable Diet among Children Aged 6–23 Months: A National and Subnational Inequality Assessment in Bangladesh
Source: Int J Environ Res Public Health. 2023 Jan 21;20(3):2010. doi: 10.3390/ijerph20032010 (PMC9915340; doi:10.3390/ijerph20032010)
Supplement: Supplementary file 1 [file ijerph-20-02010-s001.zip › ijerph-2098741-supplementary.pdf]

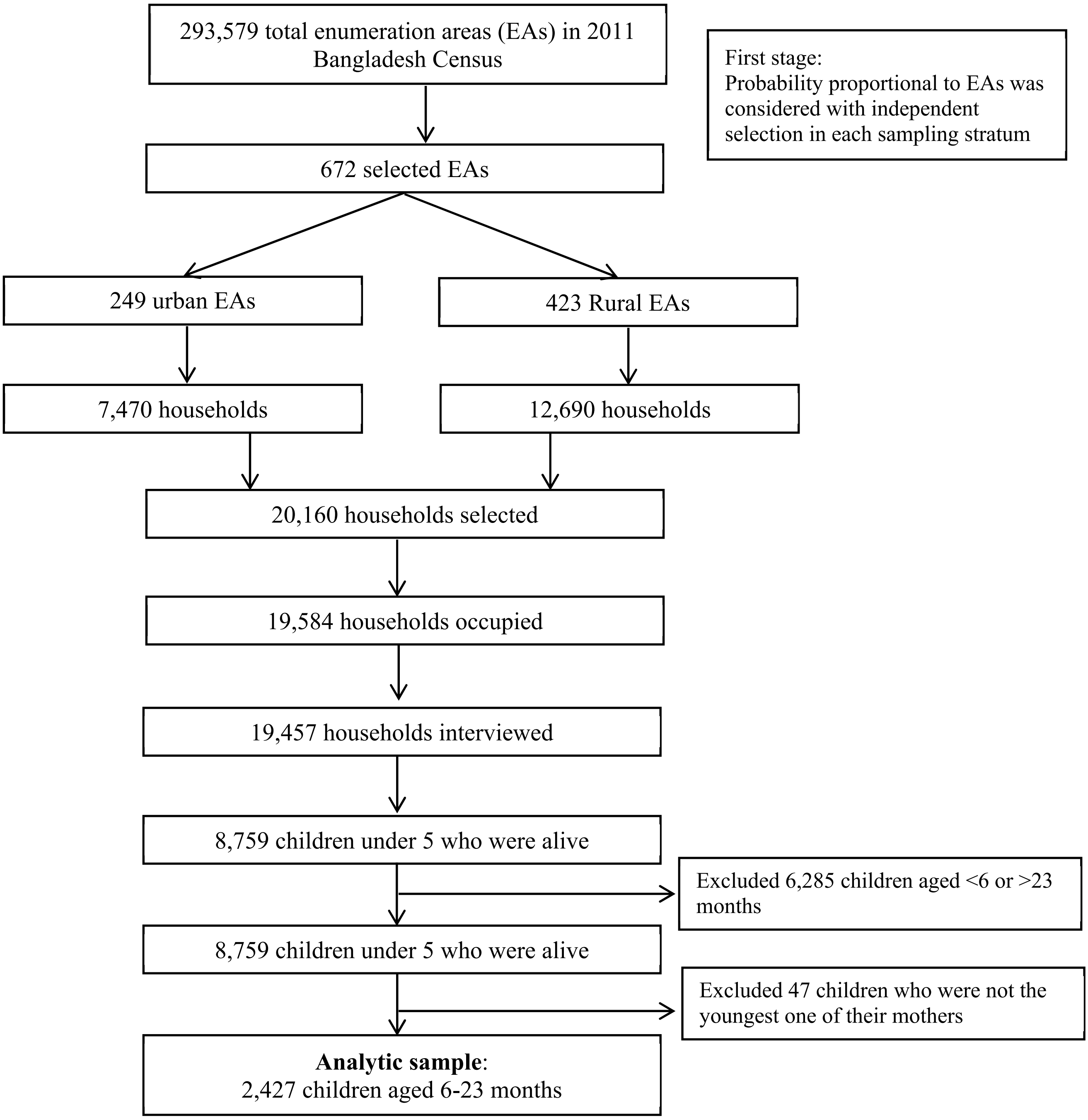

Figure S1: Participant selection.

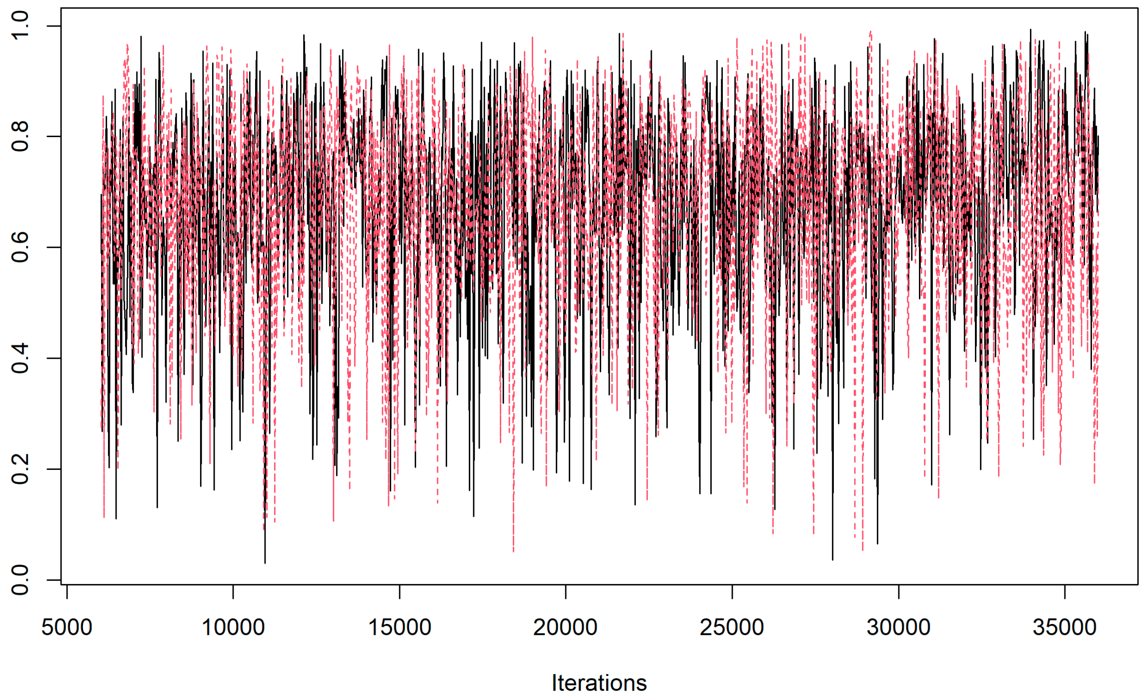

Figure S2: Trace plot for the predicted prevalence of minimum acceptable diet among Bangladeshi children in 2030.

Table S1: Complementary food groups.

**Food group**	**Contains**
Cereal and grains	Grains (bread, rice, noodles, porridge or other foods made from grains), white or pale starchy roots, tubers, and plantains
Dairy products	Cheese, infant formula, milk (powdered or fresh animal milk), yogurt
Flesh foods	Fish (fresh, dried fish or shellfish), meat (including beef, lamb, goat, chicken or duck), organ meats (including liver, kidney, heart or other organs)
Fruits and vegetables- Rich in Vitamin A	Dark green leafy vegetables; pumpkin, carrots, squash, or sweet potatoes that are yellow or orange inside; ripe mangoes; papayas; and vitamin A-rich fruits
Other fruits and vegetables	
Legumes and nuts	Any foods made from beans, nuts, lentils, peas, seeds
Breastmilk	
Eggs	

Table S2: Prevalence of minimum diet diversity, minimum diet frequency, and minimum acceptable diet among Bangladeshi children aged 6-23 months by household socioeconomic status.

	Prevalence (95% confidence intervals)					
	**Minimum diet diversity**		**Minimum meal frequency**		**Minimum acceptable diet**	
	**Poorest quintile**	**Richest quintile**	**Poorest quintile**	**Richest quintile**	**Poorest quintile**	**Richest quintile**
National	26.7 (22.5-31.2)	54.9 (49.5-60.2)	77.4 (73.6-80.9)	86 (81.4-89.6)	25.3 (21.3-29.8)	49.8 (44.8-54.9)
Place of residence						
Urban	30.5 (19.9-43.7)	55.1 (48.0-62.0)	78 (68.2-85.4)	87.7 (82.5-91.5)	29.2 (18.9-42.1)	49.6 (43.6-55.6)
Rural	26.2 (21.8-31.1)	54.6 (46.2-62.7)	77.4 (73.2-81.1)	83.2 (73.8-89.7)	24.8 (20.5-29.7)	50.2 (41.1-59.3)
Regions						
Barishal	18.8 (11.1-30.1)	67.6 (41.4-86.1)	74.2 (59.8-84.7)	89.4 (68.8-97.0)	18.1 (10.6-29.2)	61.9 (35.8-82.6)
Chattogram	11.7 (6.0-21.7)	53.4 (44.2-62.3)	66 (54.0-76.3)	80.6 (69.6-88.2)	12.9 (6.8-23.1)	48.9 (38.9-59.0)
Dhaka	37.9 (25.1-52.7)	53.6 (44.1-62.9)	77.5 (65.3-86.3)	84.2 (76.0-90.0)	37.9 (25.1-52.7)	46.1 (37.8-54.7)
Khulna	28.1 (13.6-49.3)	49.7 (34.1-65.3)	92.9 (76.3-98.2)	94.9 (84.6-98.4)	28.1 (13.6-49.3)	48.5 (32.8-64.4)
Mymensingh	25.6 (14.4-41.3)	69.3 (43.4-86.9)	81.5 (73.6-87.5)	97.6 (84.2-99.7)	24.4 (13.6-39.9)	66.9 (42.2-84.9)
Rajshahi	27.9 (16.0-43.9)	50.6 (33.3-67.7)	71.3 (56.5-82.7)	92.3 (73.7-98.1)	25.2 (13.9-41.2)	53.3 (35.8-70.1)
Rangpur	39.6 (30.5-49.5)	71.4 (49.8-86.3)	85.8 (76.1-91.9)	97.2 (81.2-99.6)	36.2 (26.9-46.7)	71.5 (51.7-85.5)
Sylhet	19.7 (12.7-29.2)	55.6 (42.2-68.2)	75.4 (63.3-84.4)	90.1 (78.4-95.8)	16.2 (10.4-24.3)	51.2 (38.2-64.2)

Table S3: Prevalence of minimum diet diversity, minimum diet frequency, and minimum acceptable diet among Bangladeshi children aged 6-23 months by maternal education.

	Prevalence (95% confidence intervals)					
	**Minimum diet diversity**		**Minimum meal frequency**		**Minimum acceptable diet**	
	**No educated**	**Higher educated**	**No educated**	**Higher educated**	**No educated**	**Higher educated**
National	18.5 (12.7-26.1)	56 (50.7-61.1)	74.1 (65.4-81.2)	89.4 (85.5-92.3)	16.3 (11.0-23.3)	52.4 (47.3-57.5)
Place of residence						
Urban	19.0 (9.0-35.6)	63.9 (55.3-71.6)	70.6 (52.6-83.8)	90.6 (84.9-94.3)	16.8 (7.8-32.4)	58.1 (49.7-66.0)
Rural	18.3 (11.8-27.4)	51.8 (45.2-58.3)	75.4 (65.2-83.3)	88.7 (83.2-92.5)	16.1 (10.2-24.5)	49.4 (43.1-55.8)
Regions						
Barishal	9.6 (1.5-43.0)	34.1 (27.9-41.0)	54.8 (19.9-85.6)	76.5 (69.6-82.2)	9.6 (1.5-43.0)	46.6 (32.4-61.3)
Chattogram	19.3 (7.7-40.4)	37.9 (32.7-43.3)	76.1 (57.3-88.3)	76.3 (71.8-80.2)	17 (6.8-36.5)	53.4 (43.2-63.3)
Dhaka	10.6 (3.4-28.7)	40.3 (35.1-45.7)	69.6 (49.2-84.4)	81.6 (76.5-85.8)	10.6 (3.4-28.7)	46 (34.6-57.7)
Khulna	16.4 (2.1-64.1)	36.8 (30.8-43.3)	64.4 (24.0-91.2)	87.3 (81.9-91.2)	16.4 (2.1-64.1)	47.8 (32.5-63.4)
Mymensingh	22.5 (9.7-44.0)	36.7 (29.1-45.0)	78.8 (53.7-92.3)	86.3 (82.5-89.4)	18 (7.1-38.5)	60.8 (43.0-76.1)
Rajshahi[a]	NA	35.5 (29.5-41.9)	68.1 (34.8-89.6)	77.9 (71.3-83.4)	NA	55.9 (40.0-70.6)
Rangpur	41.4 (19.9-66.8)	47.2 (41.4-53.0)	100	86 (80.4-90.2)	41.4 (19.9-66.8)	63.4 (50.7-74.5)
Sylhet	29.9 (15.4-50.1)	31.6 (26.2-37.5)	73.5 (55.8-85.9)	78.1 (71.8-83.3)	20.2 (10.5-35.2)	57.8 (43.0-71.3)

Note: [a]The number of samples in non-educated mother category for minimum diet diversity and minimum acceptable diet was insufficient to perform inequality analysis.

Table S4: Proportion of children consumed different food groups by household socioeconomic status and inequality assessment at the national level.

Food groups	Prevalence (95% CI)			Socioeconomic inequality	
	National	Poorest quintile	Richest quintile	SII (95% CI)	RII (95% CI)
Breastmilk	93.8 (92.6-94.9)	97.6 (95.8-98.6)	87.5 (83.4-90.7)	-12.0 (-16.4 to -7.6)***	0.88 (0.83 - 0.92)***
Cereal and grains	89.5 (88.0-90.8)	89.3 (86.1-91.9)	93.1 (90.0-95.2)	3.0 (-1.6 to 7.6)	1.03 (0.98 - 1.09)
Legumes and nuts	22.5 (20.6-24.5)	19.8 (16.3-23.8)	32.1 (27.5-37.2)	13.6 (7.0 to 20.2)***	1.82 (1.29 - 2.35)***
Dairy products	32.1 (29.9-34.5)	19.3 (15.5-23.7)	46.2 (40.7-51.8)	29.9 (22.5 to 37.3)***	2.59 (1.95 - 3.24)***
Flesh foods[1]	55.4 (53.2-57.6)	55.8 (51.2-60.4)	57.4 (52.1-62.5)	2.8 (-5.0 to 10.6)	1.05 (0.90 - 1.20)
Eggs	41.7 (39.4-44.0)	30.4 (26.1-35.1)	56.7 (50.9-62.4)	26.7 (18.9 to 34.4)***	1.92 (1.54 - 2.30)***
Fruits and vegetables					
Rich in Vitamin A	39.1 (36.8-41.6)	36.1 (31.6-40.8)	47.5 (42.3-52.8)	9.0 (1.1 to 16.9)*	1.26 (1.00 - 1.51)
Other fruits and vegetables	28.1 (25.9-30.3)	19.5 (15.9-23.8)	34.9 (29.5-40.8)	16.7 (9.1 to 24.3)***	1.81 (1.32 - 2.30)***

Note: [1]Flesh food includes meat, poultry, fish, and shellfish (and organ meats);
CI, Confidence intervals; SII, Slope index of inequality; RII, relative index of inequality.

Table S5: Proportion of children consumed different food groups by maternal education and inequality assessment at the national level.

Food groups	Prevalence (95% CI)			Socioeconomic inequality	
	National	No educated	Higher educated	SII (95% CI)	RII (95% CI)
Breastmilk	93.8 (92.6-94.9)	95.8 (90.9-98.1)	91.9 (88.5-94.4)	-3.2 (-7.0 to 0.6)	0.97 (0.93 - 1.01)
Cereal and grains	89.5 (88.0-90.8)	88.6 (80.6-93.6)	93.7 (90.7-95.8)	6.5 (1.3 to 11.6)*	1.08 (1.01 - 1.14)***
Legumes and nuts	22.5 (20.6-24.5)	16.7 (11.1-24.3)	26.3 (22.1-31.0)	9.0 (2.4 to 15.5)**	1.48 (1.05 - 1.90)***
Dairy products	32.1 (29.9-34.5)	19.6 (13.4-27.6)	45.9 (40.8-51.1)	27.1 (19.4 to 34.9)***	2.36 (1.74 - 2.98)***
Flesh foods[1]	55.4 (53.2-57.6)	35.2 (26.9-44.6)	61.6 (56.0-66.9)	15.8 (7.4 to 24.2)***	1.34 (1.13 - 1.54)***
Eggs	41.7 (39.4-44.0)	23.7 (17.0-32.1)	56.6 (51.1-61.9)	28.6 (20.5 to 36.6)***	2.02 (1.60 - 2.44)***
Fruits and vegetables					
Rich in Vitamin A	39.1 (36.8-41.6)	28.8 (21.3-37.8)	50 (45.2-54.9)	17.2 (9.5 to 24.8)***	1.55 (1.24 - 1.87)***
Other fruits and vegetables	28.1 (25.9-30.3)	24 (16.9-33.0)	37.9 (33.0-43.0)	20.1 (12.4 to 27.8)***	2.05 (1.46 - 2.64)***

Note: [1]Flesh food includes meat, poultry, fish, and shellfish (and organ meats);
CI, Confidence intervals; SII, Slope index of inequality; RII, relative index of inequality.

Table S6: Socioeconomic and education-based relative inequality in minimum diet diversity, minimum diet frequency and minimum acceptable diet among Bangladeshi children aged 6-23 months.

**Relative index of inequality**	**Socioeconomic inequality**			**Education-based inequality**		
	Minimum diet diversity	Minimum meal frequency	Minimum acceptable diet	Minimum diet diversity	Minimum meal frequency	Minimum acceptable diet
National	2.22 (1.75-2.70)***	1.11 (1.03-1.20)***	2.10 (1.64-2.56)***	2.53 (1.93-3.13)***	1.18 (1.08 - 1.28)***	2.53 (1.90-3.16)***
Place of residence						
Urban	2.41 (1.55-3.26)***	1.24 (1.07-1.41)***	2.26 (1.42-3.09)***	2.74 (1.77-3.70)***	1.21 (1.01-1.40)***	3.10 (1.91-4.29)***
Rural	2.06 (1.52-2.60)***	1.07 (0.97-1.18)	2.04 (1.47-2.60)***	2.32 (1.63-3.00)***	1.17 (1.05-1.29)***	2.26 (1.56-2.96)***
Regions						
Barishal	3.09 (0.82-5.36)	1.11 (0.83-1.40)	3.15 (0.77-5.53)	3.36 (1.27-5.45)**	1.26 (0.91-1.61)	3.77 (1.19-6.35)**
Chattogram	3.76 (1.75-5.76)***	1.28 (1.05-1.52)***	3.79 (1.60-5.98)*	1.92 (0.82-3.01)	1.09 (0.88-1.30)	2.28 (0.92-3.63)
Dhaka	1.87 (1.01-2.74)***	1.07 (0.90-1.23)	1.46 (0.78-2.14)	2.34 (1.21-3.48)***	1.31 (1.03-1.59)***	2.12 (1.01-3.23)***
Khulna	2.19 (0.99-3.39)	1.05 (0.88-1.21)	2.27 (0.99-3.56)	2.79 (1.01-4.58)**	1.14 (0.95-1.34)	2.71 (0.93-4.48)
Mymensingh	2.56 (0.94-4.18)	1.16 (1.00-1.32)	2.58 (0.96-4.20)	2.97 (1.08-4.86)**	1.19 (1.01-1.37)***	3.00 (1.08-4.91)**
Rajshahi[a]	2.31 (0.92-3.69)	1.35 (1.04-1.67)***	2.73 (1.06-4.40)**	NA	1.45 (1.04-1.85)***	NA
Rangpur	1.66 (0.93-2.39)	1.13 (0.95-1.31)	1.89 (1.02-2.75)***	2.45 (1.24-3.67)***	0.94 (0.80-1.08)	2.22 (1.13-3.31)***
Sylhet	4.03 (1.70-6.37)**	1.20 (0.95-1.45)	3.84 (1.51-6.18)**	5.28 (2.09-8.46)**	1.09 (0.89-1.28)	5.23 (1.79-8.67)**

Note: ***p<0.001; **p<0.01; *p<0.05

[a]The number of samples in non-educated mother category for minimum diet diversity and minimum acceptable diet was insufficient to perform inequality analysis.

Table S7: Determinants of minimum acceptable diet among Bangladeshi children 6-23 months (mixed-effects models).

Characteristics	Odds ratio (95% confidence intervals)			
	**Fixed-effect crude model**	**Mixed-effects model[a]**		
	**Model 1[b]**	**Model 2[c]**	**Model 3[d]**	**Model 4[e]**
Child characteristics				
Age group (months)				
6-11 (ref.)	1.00	1.00	1.00	
12-17	**2.42 (1.96-3.00)**	**2.65 (1.85-3.78)**	**2.73 (1.89-3.96)**	**2.78 (1.90-4.05)**
18-23	**2.56 (2.06-3.17)**	**2.85 (1.94-4.18)**	**2.98 (2.00-4.42)**	**3.00 (2.01-4.50)**
Sex				
Boys (ref.)	1.00	1.00	1.00	
Girls	1.08 (0.91-1.27)	1.08 (0.89-1.31)	1.09 (0.89-1.32)	1.09 (0.90-1.33)
Order of birth				
First	**1.28 (1.06-1.56)**	1.12 (0.87-1.44)	1.14 (0.88-1.48)	1.15 (0.88-1.50)
Second (ref.)	1.00	1.00	1.00	
Third or higher	0.85 (0.69-1.06)	1.04 (0.80-1.34)	1.05 (0.80-1.37)	1.05 (0.80-1.37)
Underweight				
No (ref.)	1.00	1.00	1.00	
Yes	0.84 (0.67-1.04)	1.06 (0.82-1.36)	1.09 (0.84-1.42)	1.09 (0.83-1.42)
Mother's characteristics				
Mother's age (years)				
15-20 (<20) (ref.)	1.00	1.00	1.00	
20-35	1.07 (0.86-1.33)	1.02 (0.77-1.35)	0.98 (0.73-1.32)	0.99 (0.74-1.34)
35-49 (≥35)	1.12 (0.77-1.64)	1.27 (0.76-2.11)	1.21 (0.72-2.06)	1.22 (0.71-2.08)
Mother's educational status				
Not educated (ref.)	1.00	1.00	1.00	
Primary educated	**1.68 (1.06-2.65)**	1.57 (0.94-2.61)	1.58 (0.93-2.67)	1.59 (0.93-2.70)
Secondary educated	**2.60 (1.67-4.04)**	**2.32 (1.36-3.98)**	**2.25 (1.30-3.90)**	**2.23 (1.28-3.88)**
Higher educated	**5.70 (3.58-9.05)**	**4.49 (2.29-8.80)**	**4.02 (2.06-7.84)**	**3.87 (1.97-7.60)**
Mother's working status				
Not working (ref.)	1.00	1.00	1.00	
Currently working	1.10 (0.92-1.30)	**1.31 (1.06-1.61)**	**1.41 (1.12-1.77)**	**1.35 (1.07-1.71)**
Access to mass-media				
Not at all (ref.)	1.00	1.00	1.00	
Yes (at least to some extent)	**1.86 (1.55-2.22)**	**1.34 (1.07-1.68)**	1.23 (0.97-1.57)	1.22 (0.95-1.56)
Number of ANC visit				
No ANC visit (ref.)	1.00	1.00	1.00	
1-3 visits	**1.88 (1.27-2.78)**	**1.57 (1.01-2.44)**	**1.54 (0.98-2.43)**	1.49 (0.94-2.36)
4+ visits	**4.03 (2.73-5.94)**	**2.80 (1.68-4.68)**	**2.67 (1.59-4.51)**	**2.51 (1.49-4.23)**
Household level				
Wealth quintile				
Q1 (Poorest) (ref.)	1.00		1.00	
Q2	**1.51 (1.15-1.98)**		**1.33 (0.96-1.85)**	1.39 (0.99-1.95)
Q3	**1.50 (1.13-1.99)**		**1.05 (0.74-1.49)**	1.12 (0.78-1.61)
Q4	**1.89 (1.44-2.48)**		**1.24 (0.87-1.77)**	1.35 (0.93-1.97)
Q5 (Richest)	**3.43 (2.62-4.49)**		**1.87 (1.22-2.86)**	**2.07 (1.29-3.33)**
Contextual factors				
Place of residence				

Rural (ref.)	1.00			
Urban	1.48 (1.25-1.76)			1.08 (0.85-1.38)
Regions				
Barishal	0.83 (0.57-1.20)			1.11 (0.71-1.75)
Chattogram	0.94 (0.67-1.32)			1.12 (0.74-1.70)
Dhaka	1.03 (0.74-1.45)			1.03 (0.68-1.58)
Khulna (ref.)	1.00			
Mymensingh	0.93 (0.65-1.33)			1.27 (0.81-1.97)
Rajshahi	0.99 (0.69-1.43)			1.11 (0.71-1.73)
Rangpur	**1.51 (1.06-2.15)**			**1.78 (1.12-2.84)**
Sylhet	0.79 (0.56-1.11)			**1.04 (0.67-1.62)**
**Random-effects parameter: Variance (SE)**				
Community level		0.17 (0.11)	0.17 (0.11)	0.15 (0.11)
Household level		0.12 (0.70)	0.29 (0.76)	0.37 (0.79)
Likelihood Ratio test[f]				
Chi-square statistic		4.04	3.99	3.03
P value		0.1325	0.1359	0.2199

Note: SE, standard error.
[a]Mixed-effects model included random intercept and fixed slope.
[b]Model 1, unadjusted model.
[c]Model 2 included only children's characteristics and their mother's characteristics.
[d]Model 3 further included household-level characteristics.
[e]Model 4 additionally included community-level variables (contextual factors)
[f]The likelihood ratio tests were performed to compare fixed effect model with a random-effects model. Based on the results on likelihood ratio tests, estimates of fixed effect logistic regression models were preferred than mixed-effects models. The results of fixed effect logistic regression models are presented in Table 3.

Table S8: Projected prevalence of minimum acceptable diet among Bangladeshi children aged 6-23 months (2020-2030).

	Percentage (95% credible intervals)			
	**2020**	**2022**	**2025**	**2030**
National	42.5 (21.6-63.4)	48.3 (22.5-74.1)	56.6 (21.8-85.6)	67.9 (21.1-95.6)
Wealth-quintile				
Poorest	29.4 (21.8-38.8)	34.9 (24.8-46.5)	43.9 (30.2-58.7)	59.1 (39.8-76.3)
Poorer	35.9 (26.8-45.3)	41.9 (30.6-53.2)	51.2 (36.7-65.2)	65.9 (46.1-80.9)
Middle class	43.1 (33.1-53.3)	49.3 (37.0-61.2)	58.5 (43.1-72.5)	72.1 (53.4-85.7)
Richer	48.7 (38.5-58.6)	54.9 (42.8-65.9)	63.8 (49.0-75.6)	76.4 (59.7-87.2)
Richest	55.9 (46.2-64.9)	61.9 (51.0-72.1)	70.2 (57.7-81.1)	81.2 (67.0-90.7)
Mother’s educational status				
Not educated	19.6 (8.9-31.8)	22.9 (9.4-38.9)	28.4 (9.5-52.5)	38.7 (9.7-72.8)
Primary educated	30.7 (17.1-48.3)	34.8 (17.0-56.3)	41.3 (17.7-68.8)	51.9 (18.7-84.5)
Secondary educated	36.3 (20.5-56.4)	40.7 (21.1-64.1)	47.2 (22.1-76.2)	57.5 (22.2-89.2)
Higher educated	58.8 (42.0-76.3)	62.9 (42.6-82.6)	68.5 (42.4-88.9)	76.1 (43.7-95.2)

Note: Projections were developed based on summary data from BDHS 2011, BDHS 2014 and BDHS 2017-18. Summarized data on the proportion of MAD in year 2011 and 2014 were obtained from the BDHS reports.
